# S-Glutathionylation of Protein Disulfide Isomerase Regulates Estrogen Receptor **α** Stability and Function

**DOI:** 10.1155/2012/273549

**Published:** 2012-05-13

**Authors:** Ying Xiong, Yefim Manevich, Kenneth D. Tew, Danyelle M. Townsend

**Affiliations:** ^1^Department of Cell and Molecular Pharmacology and Experimental Therapeutics, Medical University of South Carolina, Charleston, SC 29425, USA; ^2^Department of Pharmaceutical and Biomedical Sciences, Medical University of South Carolina, Charleston, SC 29425, USA

## Abstract

S-Glutathionylation of cysteine residues within target proteins is a posttranslational modification that alters structure and function. We have shown that S-glutathionylation of protein disulfide isomerase (PDI) disrupts protein folding and leads to the activation of the unfolded protein response (UPR). PDI is a molecular chaperone for estrogen receptor alpha (ER*α*). Our present data show in breast cancer cells that S-glutathionylation of PDI interferes with its chaperone activity and abolishes its capacity to form a complex with ER*α*. Such drug treatment also reverses estradiol-induced upregulation of c-Myc, cyclinD1, and P21^Cip^, gene products involved in cell proliferation. Expression of an S-glutathionylation refractory PDI mutant diminishes the toxic effects of PABA/NO. Thus, redox regulation of PDI causes its S-glutathionylation, thereby mediating cell death through activation of the UPR and abrogation of ER*α* stability and signaling.

## 1. Introduction

Glutathione S-transferase pi (GSTP) is a biomarker protein in drug-resistant solid epithelial tumors, including ovarian, breast, liver, pancreatic, lung, and lymphoma [1]. In some cases, GSTP can be the most abundant protein in the tumor and, consequently, has the potential to serve as an important drug target [2–4]. One therapeutic approach has been to develop prodrugs that are substrates for GSTP and become cytotoxic when liberated in cancer cells, yet exhibit diminished activation/toxicity in normal tissue. PABA/NO **(**O^2^-[2,4-dinitro-5-[4-(N-methylamino) benzoyloxy]phenyl] 1-(N,N-dimethylamino) diazen-1-ium-1,2-diolate) [5] is a GSTP-activated prodrug that releases high levels of nitric oxide (NO) at physiological pH. This reaction results in the formation of a Meisenheimer-complex intermediate and subsequently the leaving group of the reaction generates two molecules of NO. Elevated NO levels lead to cytotoxic effects by forming RNS/ROS intermediates that can alter protein function directly through posttranslational modifications on redox sensitive cysteine residues (S-nitrosylation, P-SNO or S-glutathionylation, P-SSG) [3, 6].

Prior studies have shown that protein disulfide isomerase (PDI) is a molecular target of PABA/NO treatment in cancer cells [2, 5, 7–9]. PDI is the most abundant chaperone/isomerase in the endoplasmic reticulum and plays a pivotal role in protein folding through isomerase and chaperone activity. The active site cysteine residues are S-glutathionylated (PDI-SSG) following PABA/NO treatment. The functional consequences are reduced isomerase activity, accumulation of unfolded/misfolded proteins, and activation of the unfolded protein response (UPR). At least one mechanism of action for PABA/NO can be attributed to UPR-induced cell death.

PDI shares homology with the estrogen-binding domain of the estrogen receptor alpha (ER*α*) [10]. Despite the KDEL sequence present in the C-terminus that retains PDI in the endoplasmic reticulum (ER), PDI is also found in non-ER locations [11]. With immune-affinity, steroid-affinity and site-specific DNA-affinity chromatography, Landel et al. have found that in extracts from MCF-7 cells and ER*α*-expressing CHO (CHO-ER*α*) cells ER*α* forms complexes with PDI, Hsp70, and two unidentified proteins [12]. Moreover, PDI enhances the binding of purified ER*α* to the estrogen response element (ERE) in a dose-dependent manner, suggesting that this accessory protein may be required for optimal interactions of ER*α* with EREs [12, 13]. Interestingly, PDI does not interact with estrogen receptor *β* (ER*β*) [10]. The DNA-binding domain and Hinge domain of ER*α* are the minimal structures sufficient for PDI-ER*α* interactions [13]. However, information regarding the corresponding domain(s) of PDI involved in this interaction is not presently available. The presence of estrogen neither is required for PDI-ER*α* interaction [13] nor has any effect on the stoichiometry of PDI association with ER*α* [12]. The PDI-ER*α* interaction may protect ER*α* from oxidation and alter ER*α* conformation so that the susceptibility of ER*α* to different proteases is altered [13].

**Figure 1:**
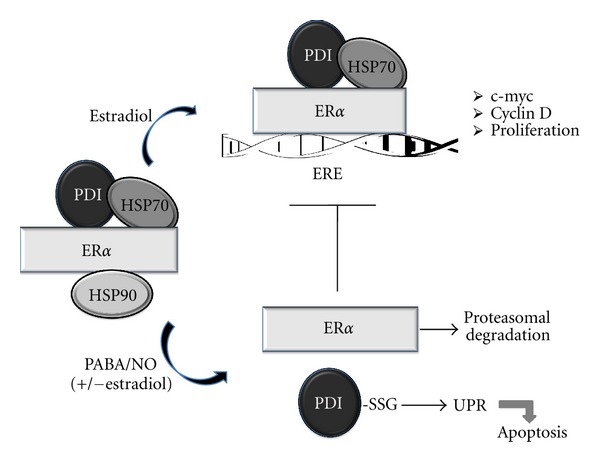
Model of the chaperone activity of PDI with ER*α*. S-Glutathionylation of PDI can interfere with protein folding, prevent protein interaction with ER*α*, and plausibly destabilize the receptor and subsequent ER*α* signaling.

The present study evaluates the efficacy of PABA/NO in ER*α*-positive breast cancer cells and interrogates the impact of S-glutathionylation of PDI on its chaperone activity, specifically with ER*α*. We hypothesize that S-glutathionylation of PDI not only blunts protein folding but also prevents protein interaction with ER*α*, destabilizing the receptor and subsequent ER*α* signaling (see scheme in Figure 1).

## 2. Material and Methods

### 2.1. Reagents

 Reduced glutathione (GSH), oxidized glutathione (GSSG), and the 3-(4,5-dimethylthiazol-2-yl)-2,5-diphenyl tetrazolium bromide (MTT) reagent, and *β*-estradiol were purchased from Sigma (St. Louis, MO). Antibodies were purchased from the following sources: anti-PDI (Affinity BioReagents); anti-glutathionylation (Virogen), anti-actin (Abcam), anti-FLAG (Sigma), anti-ER*α*/anti-ER*β* (Millipore), and anti-c-Myc/anti-Cyclin D1/anti-p21 (Cell Signaling). PABA/NO (O^2^-[2,4-dinitro-5-[4-(N-methylamino) benzoyloxy] phenyl]1-(N,N-dimethylamino) diazen-1-ium-1,2-diolate) was provided by Dr. Larry Keefer, Chemistry Section, Laboratory of Comparative Carcinogenesis, NCI at Frederick (MD).

### 2.2. Expression and Purification of His-Tagged Recombinant PDIs

His-tagged recombinant human PDI in the bacterial expression vector pET-28b was provided by Dr. Lana Lee (University of Windsor, Ontario) and expressed as previously described [8, 9]. The His-tagged PDI mutant FLFL was synthesized using the QuikChange Site-Directed Mutagenesis kit (Stratagene), in which histidine 55 and 399 were changed to phenylalanines, and lysine 57 and 401 were changed to leucines, simultaneously. All mutations were verified by DNA sequencing.

Purified plasmids were transformed into *Escherichia coli* BL21(DE3)pLysS strain, which were then induced with 1 mM IPTG at 37°C for 3 h and pelleted at 6000 rpm for 15 min at 4°C. Cells were lysed by sonication in lysis buffer (20 mM NaH_2_PO_4_, 500 mM NaCl). The lysates were centrifuged at 13,000 rpm for 30 min at 4°C and the pellets were discarded. The supernatants were incubated with Ni-NTA agarose beads (QIAGEN) for 1 h at 4°C. The beads were washed three times with wash buffer (20 mM NaH_2_PO_4_, 500 mM NaCl, and 10 mM imidazole). His-PDIs were eluted with elution buffer (20 mM NaH_2_PO_4_, 500 mM NaCl, and 250 mM imidazole). The eluates were dialyzed against dialysis buffer (25 mM HEPES, pH7.3, 100 mM NaCl, 1 mM EDTA, and 1 mM DTT). Protein purity was assessed on Coomassie-stained gels. Protein concentrations were determined using the Bradford reagent with BSA as a standard.

### 2.3. Expression of FLAG-Tagged PDIs in Mammalian Cells

Full-length human PDI was amplified from a cDNA prepared from HL60 cells using the following primers: 5′-GACGGATCCATGCTGCGCCGCGCTCTGCTG-3′and 5′-GTCGAATTCTTACAGTTCATCTTTCACAGC-3′. The resulting PCR fragment was cloned into the BamHI/EcoRI sites of pCDNA3 vector for mammalian expression. A FLAG tag sequence *DYKDDDDK* was inserted between amino acids E497 and D498, upstream of the KDEL sequence [15]. FLAG-tagged PDI mutant FLFL was also generated using site-directed mutagenesis. All mutations were verified by DNA sequencing.

The human breast cancer line MCF7 was purchased from the American Type Culture Collection and grown as recommended by the supplier. Cells were maintained in *α*MEM containing 10% FCS, 100 *μ*g/mL streptomycin, 100 units/mL penicillin, and 2 mmol/L l-glutamine at 37°C in a 5% CO_2_ incubator. Expression plasmids containing FLAG-tagged PDI and FLFL mutant were transfected into MCF7 cells using GenJet reagent according the manufacturer's instruction. Cells (2 × 10^6^ per treatment group) were plated 24 h before drug treatment.

### 2.4. Fluorescent Imaging of Native and PDI Expression Vectors

To validate that overexpression of PDI (PDI-OE) does not lead to altered localization, transfected MCF7 cells (vector or PDI) were seeded onto LabTek II Chamber Slides and allowed to adhere overnight. Cells were rinsed 2X with PBS and fixed with 4% paraformaldehyde for 30 min. followed by permeabilization in 0.2% Triton X-100 in PBS. Nuclear staining was detected using 4′,6-diamidino-2-phenylindole (DAPI). Nonspecific protein binding was blocked using 1% bovine serum albumin (BSA) for 30 min. Cells were then incubated for 1 h in primary anti-PDI (vector) or anti-FLAG (PDI-OE) antibody in PBS. Unbound primary antibody was removed by rinsing the cells 3X with PBS containing 0.05% Tween-20. The cells were incubated with Oregon Green secondary antibody. Following removal of the secondary antibody, the cells were imaged by fluorescent microscopy (Nikon eclipse E800, Nikon Instr., In. Lewisville, TX using Nikon DS-U1 software v. 5.03, Photometrics, Tucson, AZ).

### 2.5. Protein Preparation

Cells were harvested and washed with phosphate-buffered saline (PBS). Cell pellets were suspended in lysis buffer (20 mM Tris-HCl, pH 7.5, 15 mM NaCl, 1 mM EDTA, 1 mM EGTA, 1% Triton X-100, 2.5 mM sodium pyrophosphate, and 1 mM *β*-glycerophosphate with freshly added protease and phosphatase inhibitors, 5 mM NaF and 1 mM Na_3_VO_4_) and incubated for 30 min on ice. Lysates were sonicated for 10 sec and centrifuged for 30 min at 10,000 g at 4°C. Protein concentrations in the supernatant were assayed with the Bradford reagent (Bio-Rad Laboratories, Hercules, CA) using IgG as a standard.

### 2.6. Immunoblot Analysis

Equivalent amounts of protein were electrophoretically resolved under nonreducing conditions on 10% SDS-polyacrylamide gels (SDS-PAGE); unmodified proteins were separated under reducing conditions. Proteins were transferred onto nitrocellulose membranes (Bio-Rad, Hercules, CA). Nonspecific binding was reduced by incubating the membrane in blocking buffer (20 mM Tris-HCl, pH 7.5, 150 mM NaCl, 0.1% Tween 20, 1 *μ*M protease inhibitors, 5 mM NaF, and 1 mM Na_3_VO_4_) containing 10% nonfat dried milk for 1 h. Membranes were incubated with the indicated antibody (blocking buffer containing 5% nonfat dried milk) at stated dilutions overnight at 4°C, washed 3x with PBS for 15 min, and incubated with the appropriate secondary antibody conjugated to horseradish peroxidase for 1 h. The membranes were washed 3 times and developed with enhanced chemiluminescence detection reagents (Bio-Rad). The blots were scanned with a BioRad ChemiDoc system and visualized with a transilluminator. The images stored in a TIFF format. The relative intensity of bands was evaluated using Quantity One software (ver. 4.5.2; Bio-Rad) and plotted as arbitrary units (a.u.) in relation to actin.

### 2.7. Spectroscopic Analysis of PDI *In Vitro*

The structure of the S-glutathionylation refractory mutant (PDI-FLFL) was evaluated and compared to the wild type (PDI-WT) by protein tryptophan fluorescence scanning [8, 16]. The scan was recorded on an F 2500 spectrofluorometer (Hitachi) using 10 × 10 × 40 mm quartz cuvette, excitation and emission slits were 2.5 and 5.0 nm, respectively. The excitation wavelength was 295 nm to minimize an effect of protein tyrosines and phenylalanines. Background spectra were subtracted from final emission of the protein. Quenching experiments were performed as follows: 1 *μ*M of PDI-WT or PDI-FLFL in 20 mM PB, pH = 7.4 was titrated with 0–5000 nM CsCl at room temperature under constant stirring. The Trp emission spectra (Ex. = 295 nm) were recorded and integrated using Felix32 standard software (PTI, NJ). All data were normalized to the emission proteins prior to addition of CsCl. Data represent the mean ±  SD for 3 independent experiments.

### 2.8. PDI Activity Assay

The enzyme activities of PDI-WT and PDI-FLFL were monitored using the turbidimetric assay of insulin disulphide reduction [17]. Briefly, 0.25 *μ*M purified human PDI was added to a solution of insulin (0–250 *μ*M) and GSH (500 *μ*M) in a 0.1 M K_2_HPO_4_ buffer containing 2 mM EDTA (pH 7.0). DTT has been shown to completely reduce the disulfide bonds and is used as a positive control. The increase in turbidity was monitored (*λ* = 630) at 30 s intervals for 30 min. Mean values and S.E. were computed for each group, *N* = 3.

### 2.9. Cytotoxicity Assays

10,000 cells were seeded in 96-well plates in 50 *μ*L medium. Increasing drug concentrations of PABA/NO were added to a final volume of 100 *μ*L and maintained in drug for 72 h. Following drug exposure, cell viability was assayed by the 3-(4,5-dimethylthiazol-2-yl)-2,5-diphenyl tetrazolium bromide (MTT) conversion assay [14]. Each drug concentration was represented in quadruplicate and three independent experiments were conducted. Mean values and S.E. were computed for each group.

### 2.10. Statistical Analysis

Experimental data presented represent the mean value ± SD for at least three independent experiments. Data were analyzed for statistically significant differences between groups with Student's *t*-test using SigmaStat 3.5 (Systat Software Inc., San Jose, CA). Differences were considered statistically significant if the *P* value was <0.05.

## 3. Results

### 3.1. S-Glutathionylation of PDI Disrupts the ER*α*-PDI Complex and Stability

**Figure 2:**
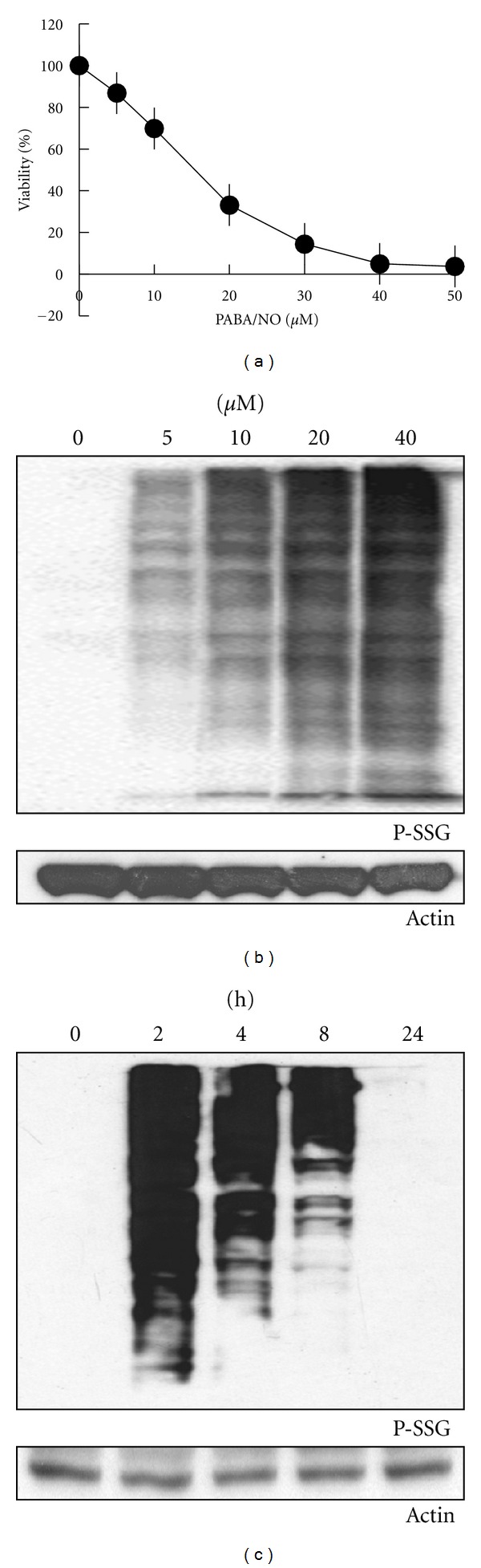
PABA/NO-induced S-glutathionylation of PDI *in vitro*. (a) MCF7 cells were seeded in a 96-well plate and treated with 0–50 *μ*M PABA/NO. Cell viability was measured at 72 h with the MTT assay [14]. Data represent the mean for 3 independent experiments ± S.D. (b) MCF7 cells were treated with DMSO or PABA/NO at the indicated concentrations for 2 h. (c) MCF7 cells were treated with DMSO or 20 *μ*M PABA/NO for the indicated times. Cell lysates (40 *μ*g) were separated and under nonreducing conditions and subjected to Western blot with anti-S-glutathionylation (P-SSG) and antiactin antibodies.

**Figure 3:**
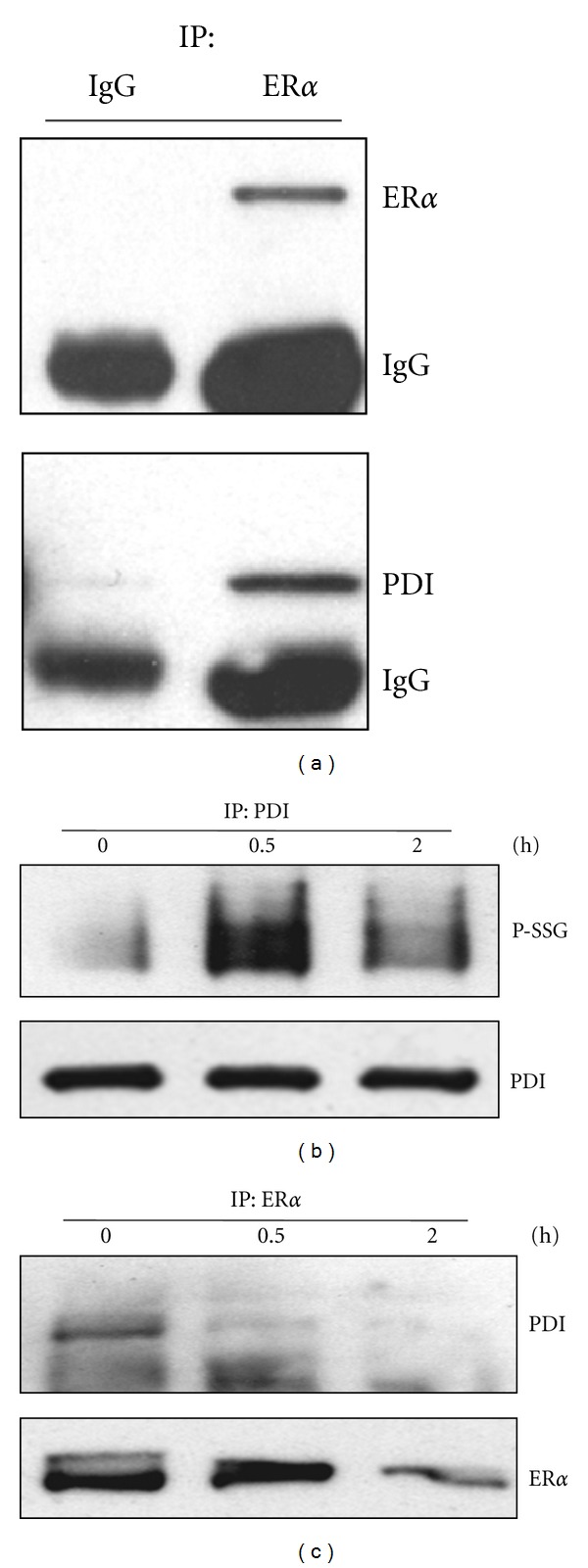
PABA/NO abrogates PDI-ER*α* interaction. (a) MCF7 cell lysates (1 mg) were incubated with anti-ER*α* antibody or control IgG at 4°C overnight. (b) MCF7 cells were treated with DMSO or 20 *μ*M PABA/NO for the indicated times. Cell lysates (300 *μ*g) were incubated with anti-PDI antibody at room temperature for 4 h. The precipitated endogenous PDI was subjected to Western blot with anti-*S*-glutathionylation (P-SSG) and anti-PDI antibodies. (c) MCF7 cells were treated with DMSO or 20 *μ*M PABA/NO for the indicated times. Endogenous ER*α* was precipitated with the anti-ER*α* antibody at 4°C overnight. The precipitates were subjected to Western blot with anti-ER*α* and anti-PDI antibodies.

Prior studies have shown that PABA/NO induces dose-dependent cell death that is concurrent with S-glutathionylation and activation of the UPR in ovarian and leukemia cancer cells [8]. Using MCF7 cells, we confirmed the presence of a dose- (Figure 2(a)) and time-dependent inverse relationship of P-SSG relative to cell death (Figure 2). Under nonstressed conditions, PDI coimmunoprecipitates with ER*α*, Figure 3(a). However, PABA/NO-induced nitrosative stress-induced S-glutathionylation of PDI abrogates the interaction within 30 min following drug treatment (Figures 3(b) and 3(c)).

### 3.2. PDI Levels Affect Drug-Induced ER*α* Proteasomal Degradation

**Figure 4:**
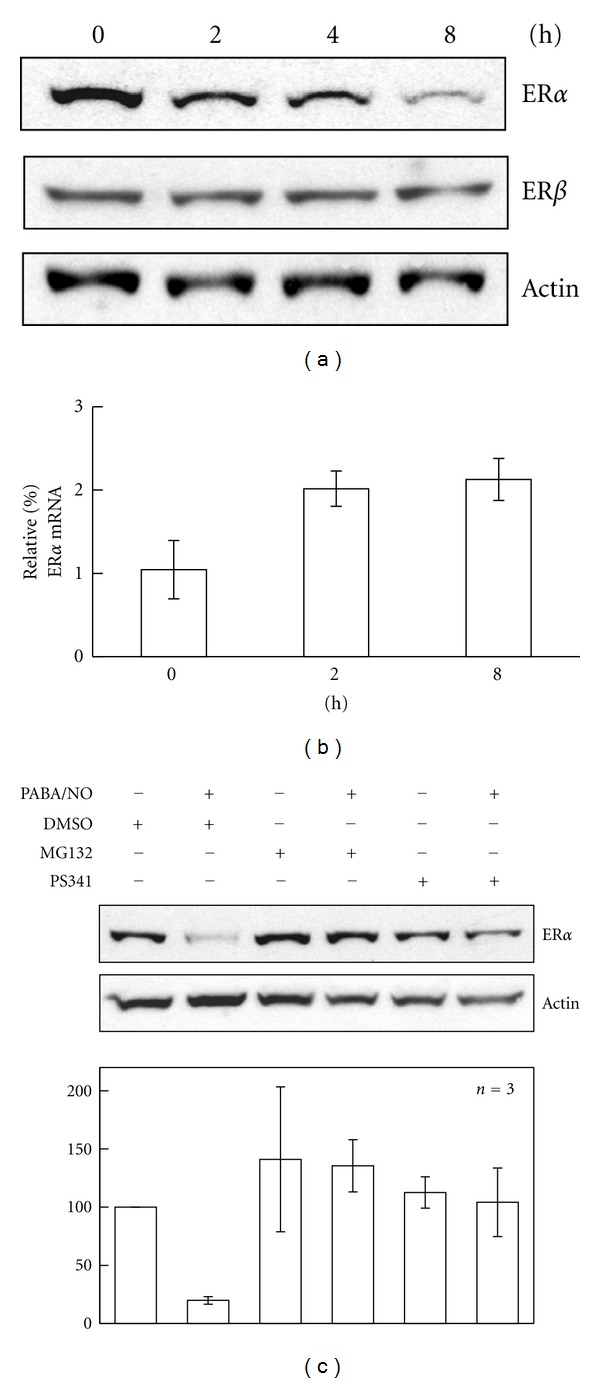
PABA/NO leads to ER*α* proteasomal degradation. (a) MCF7 cells were treated with DMSO or 20 *μ*M PABA/NO for the indicated times. Cell lysates (40 *μ*g) were subjected to Western blot with the indicated antibodies. (b) MCF7 cells were treated with DMSO or 20 *μ*M PABA/NO for the indicated times. (c) MCF7 cells were pretreated with proteasome inhibitors MG132 (1 *μ*M) or PS341 (10 *μ*M) for 2 h and then treated with 20 *μ*M PABA/NO for 6 h. Cell lysates (40 *μ*g) were subjected to Western blot with the indicated antibodies. Bar diagram showed the means and standard deviations determined from three independent experiments.

**Figure 5:**
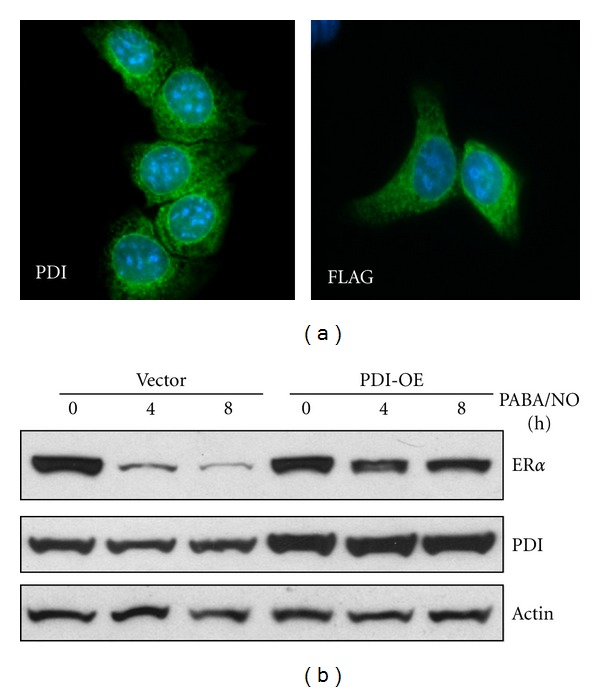
PDI expression levels affect PABA/NO-stimulated ER*α* degradation. (a) shows fluorescent images detailing similar subcellular distribution of PDI in normal and transfected cells. (b) shows Western blots of vector or PDI-transfected cells treated with PABA/NO for various times. In the transfected cells, over-expression of PDI stabilized intracellular ER*α* levels, preventing PABA/NO-induced S-glutathionylation from causing Er*α* degradation.

Chaperone functions of PDI are considered to be protective of ER*α* when exposed to either oxidative or nitrosative damage [13]. In fact, protein levels of ER*α* were diminished 81 ± 2% following treatment with PABA/NO (Figure 4(a)). To determine if ER*α* levels were decreased as a consequence of proteosomal degradation, MCF7 cells were pretreated with proteosomal inhibitors (MG132 and PS341) prior to subsequent PABA/NO exposures. Proteosomal inhibition blunted PABA/NO-induced ER*α* degradation (Figure 4(c)). To validate that PDI levels are critical to ER*α* stability, MCF7 cells were transfected with empty vector or with recombinant PDI to generate overexpressing cells.  Figure 5(a) shows fluorescent images confirming that the sub-cellular localization of transfected, overexpressed PDI (right panel) is consistent with native PDI (left panel). Comparative analysis of vector or PDI-transfected cells treated with PABA/NO (Figure 5(b)) suggested that overexpression of PDI stabilized intracellular ER*α* levels.

### 3.3. PDI-SSG Alters ER*α*-Mediated Gene Expression

**Figure 6:**
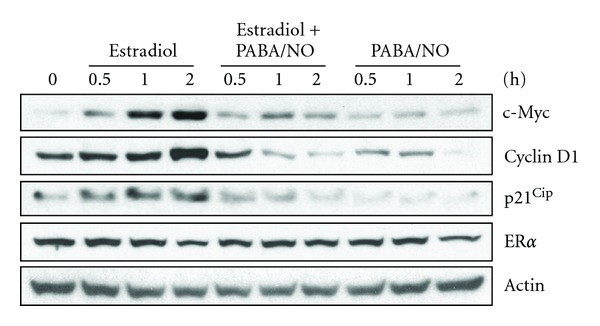
PABA/NO compromises ER*α*-mediated gene expression. MCF7 cells were treated with DMSO, 20 nM *β*-estradiol (E2), 20 *μ*M PABA/NO or both for the indicated times. Cell lysates (40 *μ*g) were subjected to Western blot with the indicated antibodies.

Previous studies have shown that estrogen stimulation can lead to activation of a range of proteins involved in cell proliferation [10]. We evaluated a cadre of such response proteins following **β**-estradiol ± PABA/NO treatments (Figure 6). PABA/NO treatment appeared to counteract the expected estrogenic effects, leading to downregulation of c-Myc, Cyclin D1, and p21. Such data are consistent with the antiproliferative and growth arrest effects of PABA/NO.

### 3.4. S-Glutathionylation Refractory Mutant PDI Decreased PABA/NO-Induced Toxicity

**Figure 7:**
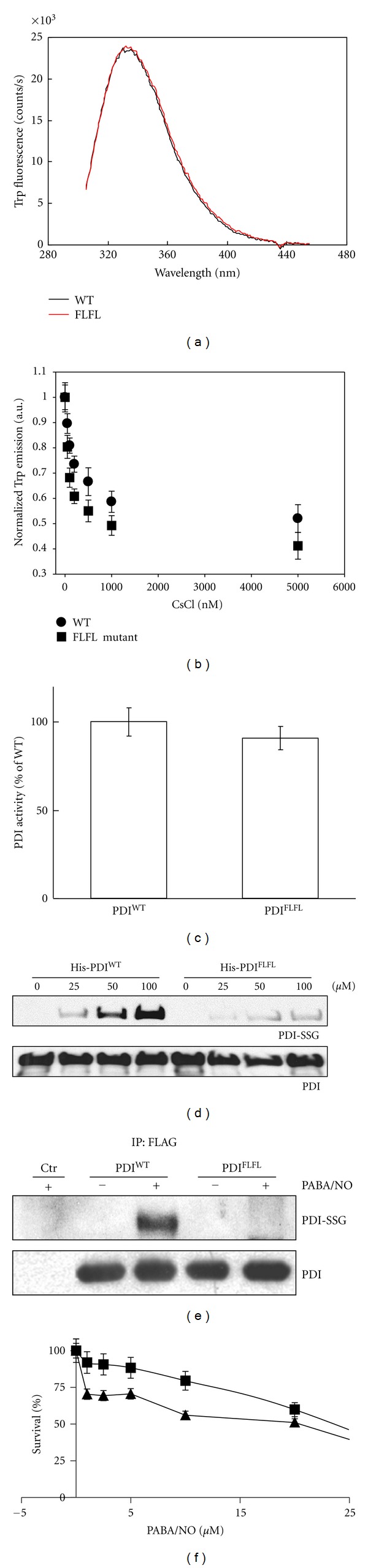
Mutations impair PABA/NO-induced S-glutathionylation of PDI. (a) Spectroscopic analysis of PDI-WT (black) and PDI-FLFL (red) was performed using trypophanyl fluorescence of the purified proteins. (b) Quenching experiments were performed to further assess protein folding for PDI-WT (●) and PDI-FLFL (■). (c) The enzymatic activity of PDI-WT and PDI-FLFL was evaluated using the insulin turbidity assay. No statistical differences were observed in the folding (a-b) or isomerase activity (c), *P* > 0.05. (d) S-glutathionylation of PDI-WT and PDI-FLFL was evaluated following drug treatment. (e) MCF7 cells overexpressing PDI-WT or PDI-FLFL were treated with DMSO or 20 *μ*M PABA/NO. Following immunoprecipitation, the samples were evaluated by Western blot with anti-S-glutathionylation and anti-PDI antibodies. (f) MCF7 cells transfected with control (▲) or PDI-FLFL (■) were seeded in a 96-well plate and treated with 0–25 *μ*M PABA/NO. Cell viability was measured at 72 h with the MTT assay [14]. Data represent the mean for 3 independent experiments ± S.D.

S-glutathionylation invariably occurs at cysteine residues found in a motif that exists in a low pKa environment [3]. Using site-directed mutagenesis we generated an S-glutathionylation refractory mutant, transfected it into host cells (PDI-FLFL), and evaluated its secondary and tertiary structure compared to PDI and PDI-SSG [8, 9]. S-Glutathionylation altered both the CD spectra and tryptophanyl fluorescence of native PDI. The trypophanyl fluorescence scans of PDI-WT and PDI-FLFL have overlapping spectra (*P* < 0.05), indicating the proteins are folded similarly (Figure 7(a)). Further validation that protein folding of PDI-FLFL was equivalent to PDI-WT was based upon the data showing quenching of the protein Trp fluorescence with CsCl (Figure 7(b)). There was no statistical difference between the PDI-WT protein and the S-glutathionylation refractory mutant (*P* < 0.05). Measurement of isomerase activity showed PDI and PDI-FLFL were essentially equivalent (Figure 7(c)). Recombinant proteins were incubated with PABA/NO and GSH for 30 min. PDI was S-glutathionylated in a dose-dependent manner, however PDI-FLFL was refractory (Figure 7(d)). PDI-WT and PDI-FLFL were transfected into MCF7 cells and treated with PABA/NO to induce S-glutathionylation and activate UPR-induced cell death. Following immunoprecipitation, PDI-WT was S-glutathionylated as detected by Western blot analysis. However, PDI-FLFL was not modified (Figure 7(e)). S-Glutathionylation of PDI is the trigger for ER*α*-PDI complex disruption and activation of the UPR. Consequently, the toxic effects of PABA/NO were diminished in MCF7 cells transfected with the S-glutathionylation-resistant mutant, PDI-FLFL (Figure 7(f)).

## 4. Discussion

ER*α* signaling is a contributing factor in the regulation of breast cancer cell proliferation. Targeting ER*α* signaling is an important and successful treatment strategy for most breast cancer patients that are ER*α* positive. In the preclinical evaluation of PABA/NO, PDI was identified as a molecular target of the drug [2, 5, 8, 18–20]. Specifically, PDI is S-glutathionylated on cysteine residues within the catalytic domains following nitrosative stress, thereby blunting isomerase activity [8, 9]. PDI has homology with the estrogen-binding domain of ER*α* and acts as a molecular chaperone that is required for ER*α*-mediated gene regulation [21]. In the present study we evaluated whether S-glutathionylation of PDI also impacts chaperone function. Our data suggest that S-glutathionylation of PDI leads to disruption of the PDI:ER*α* complex, proteosomal degradation of transcription factor, and diminished ER*α* mediated gene regulation.

PDI is organized into five domains (a, b, b′, a′, and c). There are two active sites in the catalytic domains a and a′, each having two conserved cysteine residues that cycle between oxidized (disulfide) and reduced (dithiol) states to facilitate the folding and correct disulfide bond formation of its protein substrates [22]. The crystal structure of yeast PDI suggests that the a, b, b′, and a′ domains form a twisted U shape with the catalytic domains facing each other and an internal hydrophobic surface that interacts with substrate proteins [23]. Residues 101–144 of mature PDI have significant similarity (29.5% identity) with residues 350–392 of ER*α*. Moreover, residues 163–211 of mature PDI are homologous with residues 304–349 of ER*α*. It is noteworthy that PDI segment 101–144 comprises the C-terminal part of the a domain, a short interdomain linker, and N-terminal part of the b domain, whereas segment 163–211 is exclusively located in the b domain. It would seem plausible that estrogen binding may cause a conformational change that interferes with the functional coordination between the catalytic domain and substrate-binding region, with the consequence that PDI enzymatic activity is suppressed. Accordingly, the apparent *K*_*d*_ value of PDI for estrogens such as *β*-estradiol has been determined to be 1.5–2 *μ*M [10]. The catalytic activity of PDI (measured by insulin degradation or reactivation of randomly oxidized RNase) is inhibited by estradiol *in vitro*, with an IC_50_ of approximately 100 nM [21]. As an estrogen-binding protein, PDI can serve to modulate the intracellular estrogen reservoir and slow down estrogen metabolic disposition. Moreover, under certain conditions, PDI-bound estrogen can be released and augment the transcriptional activity of ER*α* [10].

S-Glutathionylation of proteins generally occurs on cysteine residues with a low pKa [3]. Using site-directed mutagenesis we generated a mutant PDI that is S-glutathionylation refractory with isomerase activity equivalent to native PDI. Transfection of PDI-FLFL into breast cancer cells resulted in diminished PABA/NO-induced toxicity as a result of S-glutathionylation refractory PDI. Hence the triggers for abolishing the ER*α*-PDI complex and activation of the UPR were blunted.

Our present data indicate that PDI is an ER*α*-interacting partner. Previously, studies have been designed to determine whether PDI regulates ER*α* protein levels. RNAi technology has been used to knock down PDI in MCF-7 cells but interpretation of results has not always been consistent. For example, Fu et al. demonstrated that PDI knockdown is associated with downregulation of ER*α* [10], whereas Schultz-Norton et al. reported the exact opposite finding that decreased expression of PDI leads to an increase in ER*α* protein levels [13]. Both groups have used at least two different RNAi sequences, thus a precise understanding of the relationship between PDI and ER*α* stability remains undefined. The results from the present study demonstrate that PDI levels do have a direct impact on ER*α* stability. Moreover, changes in redox homeostasis induced by nitrosative/oxidative stress cause S-glutathionylation of PDI and mediate cell death through activation of the UPR and abrogation of ER*α* stability and signaling.
